# Serine-Arginine Protein Kinase 1 (SRPK1) as a Prognostic Factor and Potential Therapeutic Target in Cancer: Current Evidence and Future Perspectives

**DOI:** 10.3390/cells9010019

**Published:** 2019-12-19

**Authors:** Ilias P. Nikas, Sophie C. Themistocleous, Stavroula A. Paschou, Konstantinos I. Tsamis, Han Suk Ryu

**Affiliations:** 1 School of Medicine, European University Cyprus, 2404 Nicosia, Cyprus; S.Themistocleous@euc.ac.cy (S.C.T.); s.a.paschou@gmail.com (S.A.P.); ktsamis1981@yahoo.gr (K.I.T.); 2 Division of Endocrinology and Diabetes, “Aghia Sophia” Hospital, Medical School, National and Kapodistrian University of Athens, 11527 Athens, Greece; 3 Neurosurgical Institute, Medical School, University of Ioannina, 45500 Ioannina, Greece; 4 Department of Pathology, Seoul National University Hospital, 03080 Seoul, Korea; karlnash@naver.com

**Keywords:** serine-arginine protein kinase 1 (SRPK1), alternative splicing, TNM staging, prognosis, cancer survival, personalized medicine, chemotherapy resistance, metastasis, angiogenesis, apoptosis

## Abstract

Cancer, a heterogeneous disease composed of tumor cells and microenvironment, is driven by deregulated processes such as increased proliferation, invasion, metastasis, angiogenesis, and evasion of apoptosis. Alternative splicing, a mechanism led by splicing factors, is implicated in carcinogenesis by affecting any of the processes above. Accumulating evidence suggests that serine-arginine protein kinase 1 (SRPK1), an enzyme that phosphorylates splicing factors rich in serine/arginine domains, has a prognostic and potential predictive role in various cancers. Its upregulation is correlated with higher tumor staging, grading, and shorter survival. SRPK1 is also highly expressed in the premalignant changes of some cancers, showing a potential role in the early steps of carcinogenesis. Of interest, its downregulation in preclinical models has mostly been tumor-suppressive and affected diverse processes heterogeneously, depending on the oncogenic context. In addition, targeting SRPK1 has enhanced sensitivity to platinum-based chemotherapy in some cancers. Lastly, its aberrant function has been noted not only in cancer cells but also in the endothelial cells of the microenvironment. Although the aforementioned evidence seems promising, more studies are needed to reinforce the use of SRPK1 inhibitors in clinical trials.

## 1. Introduction

Cancer pathogenesis is driven by deregulated signaling pathways that result in uncontrolled proliferation, enhancement of angiogenesis, epithelial-mesenchymal transition (EMT), invasion, metastasis, and evasion of apoptosis. Of these hallmarks, metastasis to distant sites is the main cause of cancer death [1,2]. Each cancer is composed not only of tumor cells but also of diverse components that collectively form the tumor microenvironment. The endothelial cells and pericytes of blood vessels, cancer-associated fibroblasts (CAFs), various immune and inflammatory cells, and extracellular matrix (ECM) all interact with the tumor cells and influence cancer progression, survival, and response to therapy [3,4]. Notably, cancer is not a single disease yet appears heterogeneous among individuals (intertumor heterogeneity), also within its own tumor mass in each affected individual (intratumor heterogeneity) [5,6]. Apart from the tumor microenvironment, cancer stem cells (CSCs) are also a major area of research in the field of intratumor heterogeneity, while their presence is associated with cancer recurrence, metastasis, and resistance to chemotherapy [7].

Cancer prognosis depends on multiple factors that affect survival. Tumor staging, traditionally performed with the TNM system, is the most important prognostic factor. It refers to the size of the tumor (T), also the extent of its spread to the regional lymph nodes (N) or distant metastatic sites (M) [8,9]. Grading refers to the histologic picture of the tumor, more specifically, how closely it looks compared to the normal tissue it derives from (differentiation) [10,11]. Both the presence of distant metastases (e.g., in lungs, brain, liver, bones) and poor differentiation are associated with a dismal prognosis [9,11]. The molecular subtype of specific cancers is also critical for cancer survival. For instance, breast cancer has diverse intrinsic subtypes—luminal A and B, human epidermal growth factor receptor 2-enriched (HER2-enriched), and basal-like—that directly impact prognosis [12,13,14]. Luminal breast cancers are most commonly hormone-positive, overexpressing estrogen receptors (ER), and are associated with a better prognosis than HER2-enriched or basal-like breast cancers (BLBCs) [12,13,14,15]. BLBCs, which most commonly present with a triple-negative phenotype, have been linked with the worst prognosis and highest metastatic potential of all breast cancer molecular subtypes [13,16,17].

Alternative splicing is the process that removes introns and adds exons in various combinations resulting in multiple mRNA products hence protein transcripts. As a result, it maintains the protein diversity and cellular homeostasis [18]. The majority of human genes undergo alternative splicing [19]. Serine-arginine protein kinase 1 (SRPK1) is an enzyme that phosphorylates splicing factors rich in serine/arginine domains (SR proteins); thus, it has a central role in alternative splicing regulation [20,21]. A prototype of SR proteins is the serine/arginine-rich splicing factor 1 (SRFS1). SRPK1 gene is located on chromosome 6 and its product is overexpressed in normal pancreas and testicular germ cells, while it is underexpressed in glia [22,23,24]. Briefly, SRPK1 interacts with SR proteins (e.g., SRFS1) and regulates several normal cellular processes including various steps of RNA maturation, chromatic reorganization, cell cycle progression, and immune response [20,25]. In contrast, deregulation of the splicing machinery contributes to the pathogenesis of diseases such as frontotemporal dementia, Alzheimer’s disease, atherosclerosis, arthritis, macular degeneration, human papillomavirus infection, and cancer [2,20,25].

Accumulating evidence suggests that the aberrant function of alternative splicing is a key mechanism of carcinogenesis that is, in fact, linked with the hallmarks of cancer [1,2,19]. Of interest, splicing isoforms of a single pre-mRNA can function in opposing ways to one another, enhancing or suppressing one or more oncogenic processes such as angiogenesis, invasion, metastasis, and apoptosis [2,19]. For instance, vascular endothelial growth factor A (VEGF-A) can produce both proangiogenic and antiangiogenic isoforms; thus, a shift in the splicing machinery towards the production of the former enhances angiogenesis in cancer [26]. In fact, targeting alternative splicing could pose as an attractive treatment strategy for various cancers [18,25].

The following review examines the role of SRPK1 as a prognostic factor and potential therapeutic target in cancer and is structured as follows. First, we address the existing evidence that highlights the prognostic role of SRPK1, as shown in human samples. Second, we describe the published preclinical studies (cell lines and/or animal models) that support SRPK1 candidacy as a target for cancer treatment. Lastly, we discuss our findings and give a direction for future research.

## 2. SRPK1 as a Prognostic Factor

SRPK1 has been investigated in clinical material of various cancers, and its expression has been correlated with prognostic factors (e.g., staging, grading, and molecular subtypes) and survival. SRPK1 high expression is found in most human cancers; this is shown in the analysis of publicly available TCGA data accessed through the UALCAN website (http://ualcan.path.uab.edu/cgi-bin/Pan-cancer.pl?genenam=SRPK1) [27]. A summary of all published gene expression studies on human material that highlight the prognostic role of SRPK1 in various cancers is shown in Table 1.

Except its reported overexpression in non-small cell lung cancer (NSCLC) compared to normal lung tissue [29,30,31], evidence shows that SRPK1 expression status could be a prognostic factor in this cancer type. Gong et al. performed SRPK1 immunohistochemistry (IHC) on histology samples of NSCLC patients and noted that SRPK1 overexpression was correlated with higher staging—a significant association with all components of the TNM System (T, N, and M) was found—and shorter survival at both univariate and multivariate levels. Thus, SRPK1 overexpression was found to be an independent poor prognostic factor in NSCLC [29]. Of interest, SRSF1 was also upregulated in NSCLC and its overexpression was associated with higher staging and increased metastatic potential [56]. Testing in human samples hence showed that the SRPK1/SRSF1 axis is implicated in NSCLC progression and survival.

Similar to NSCLC, SRPK1 overexpression was also found in breast cancer clinical samples compared to normal breast tissue [22,32,35,36] and was correlated with higher grading [36], staging—once more, a significant association with all components of the TNM System (T, N, and M) was noted—and shorter survival at both univariate and multivariate levels [32]. SRRPK1 upregulation seemed to favor a specific distant metastatic pattern to the lungs and brain, rather than bones and liver [33]. Furthermore, SRPK1 expression was investigated in relation to the breast cancer molecular subtypes and resistance to chemotherapy. Van Roosmalen et al. reported that SRPK1 overexpression in ER-positive breast cancer patients correlated with shorter metastasis-free survival and higher proliferation status, as shown with Ki-67 IHC. When they sub-categorized luminal breast cancer patients into luminal A and B, they found a significant association between SRPK1 overexpression and the more aggressive of the two luminal B subtype [33]. In another study, Cheng et al. performed a data-mining study using the GEO database and showed that SRPK1 is overexpressed in highly aggressive TNBCs. In addition, they linked SRPK1 overexpression with a shorter distance-relapse free survival in HER2-negative patients treated with chemotherapy, suggesting SRPK1 might have a role in the development of resistance to therapy [34].

SRPK1 upregulation has also been described in other common epithelial cancers and has been correlated with various prognostic factors and survival. In prostate cancer, SRPK1 overexpression was noted in both cancer and its precursor, PIN (prostatic intraepithelial neoplasia), and was associated with a higher tumor stage [26,37]. Similarly, in colorectal cancer, SRPK1 was overexpressed in both cancer and its precursor, adenoma, compared to the normal colonic tissue [22,31,36,38,39,40]; its high expression was also linked with higher grade and stage colorectal cancers, presence of lymph node metastases, in addition to shorter overall and recurrence-free survival [31,36,38,39]. In stomach cancer, high SRPK1 was also found compared to the normal stomach histology [31,41,42,43,44] and was associated with higher grade, stage, larger tumor size, lymph node metastases, and shorter overall survival [41,43,44]. Liver cancer was also characterized by SRPK1 overexpression, while the latter was correlated with higher stage, larger tumor size, and shorter survival [45,46,47]. In esophageal cancer, high expression of SRPK1 was associated with higher grade, stage, the capacity to metastasize, and shorter overall survival at both univariate and multivariate levels [48].

In contrast to epithelial malignancies, the results of SRPK1 expression seemed less predictable in non-epithelial cancers. For instance, while SRPK1 was highly expressed in gliomas, this was correlated with low rather than high-grade gliomas [51]. However, SRPK1 overexpression was linked with high proliferative activity (as measured with Ki67 IHC) and shorter survival in glioblastomas (WHO grade IV gliomas) [52]. In patients with NSGCTs (non-seminomatous germ cell tumors) of the testis, low SRPK1 expression was associated with resistance to chemotherapy, whereas high SRPK1 expression characterized the tumors that responded to therapy [23]. Lastly, patients with advanced retinoblastomas, especially the ones that recurred or metastasized, were found to have low rather than high expressions of SRPK1 [55].

## 3. SRPK1 as a Potential Therapeutic Target in Cancer

**Figure 1:**
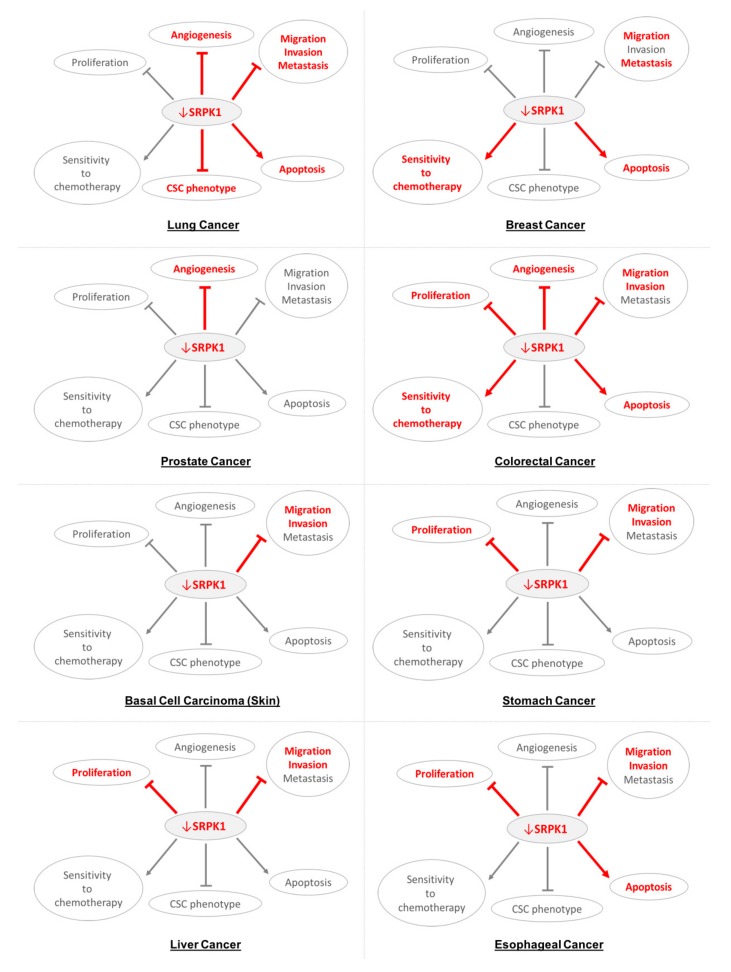

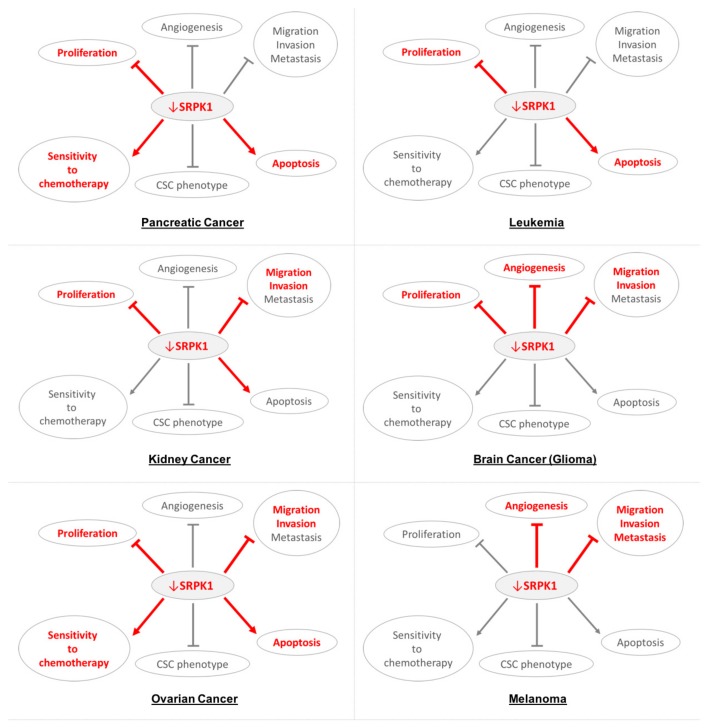
This figure shows the effect of serine-arginine protein kinase 1 downregulation (↓SRPK1) on distinct oncogenic processes, highlighting its potential role as a therapeutic target in various malignancies. Fourteen different cancer types are shown. Arrows and inhibition symbols represent promotion or suppression, respectively, of the pointed processes after SRPK1 downregulation in each one of the listed cancer types. Red color means the specific interaction between SRPK1 and the pointed process is discussed in the literature. Gray color could mean that the interaction is not described, described but not activated (e.g., ↓SRPK1 doesn’t involve proliferation, migration, and invasion in prostate cancer or proliferation in melanoma) or described with an opposite result in the literature (e.g., ↓SRPK1 enhances resistance, rather than sensitivity to chemotherapy in gliomas).

Up to date, no clinical trials targeting SRPK1 as a cancer treatment have been performed or are currently running (https://clinicaltrials.gov/). However, several preclinical studies on cell lines and animal models have tested the effect of aberrant SRPK1 expression on distinct oncogenic processes highlighting the potential role of SRPK1 as a therapeutic target in various cancers. A summary of these studies is shown in Table 2. Of interest, existing evidence supports that SRPK1 acts heterogeneously among various cancer types. Different regulatory pathways are disrupted hence distinct oncogenic processes (e.g., proliferation, angiogenesis, apoptosis) are primarily influenced depending on the cellular context [57]. Targeting SRPK1 in cancer could simultaneously affect different diverse processes in every cancer type, as shown in Figure 1, making it a potentially attractive treatment strategy. This chapter mainly focuses on the processes affected in each cancer when SRPK1 is downregulated (e.g., by inhibitors or gene silencing), while it briefly describes the effects of SRPK1 upregulation and the main pathways involved.

In NSCLC, a main oncogenic process affected by SRPK1 was reported to be the acquisition of a CSC (cancer stem cell) phenotype. SRPK1 upregulation promoted cell growth, CSCs accumulation, and the expression of various stem cell markers, while its downregulation suppressed cell growth, CSCs, and tumorigenicity [29]. Gong et al. also found that SRPK1 regulates CSCs in NSCLC by interacting with the Wnt/β-catenin pathway [29]. Other teams reported that SRPK1 interacts with the β-catenin/TCF (T-cell factor) pathway, also the GSK3-β (glycogen synthase kinase 3-β), and promotes cell growth, migration and invasion, while its downregulation suppresses metastasis and induces apoptosis [30,58]. Except for NSCLC cells, SRPK1 acts on the endothelial cells of the tumor microenvironment. WT-1 (Wilms tumor-1) interacts with the SRPK1/SRFS1 axis and regulates the alternative splicing of VEGF (vascular endothelial growth factor) [59]. VEGF alternative splicing can result either in proangiogenic or antiangiogenic isoforms, regulating angiogenesis in cancer [19]. Wagner et al. noted that, whereas SRPK1 overexpression induced the expression of proangiogenic VEGF164a isoform, its reduced expression resulted in a shift towards the expression of the antiangiogenic VEGF120, suppressing angiogenesis in vivo [59]. To summarize, SRPK1 downregulation affects both tumor epithelial cells and endothelial cells of the microenvironment, whilst it suppresses the acquisition of CSC phenotype, migration, invasion, metastasis, and angiogenesis and promotes apoptosis in preclinical NSCLC models.

In breast cancer, SRPK1-induced alternative splicing plays a crucial role in oncogenesis as well. SRPK1 regulates apoptosis by interacting with RBM4 (RNA-binding motif protein 4) and modulating the alternative splicing of IR (insulin receptor) and MCL-1 (myeloid cell leukemia-1) [35]. SRPK1 downregulation enhanced apoptosis in hormone-positive breast cancer models by reducing RBM4 phosphorylation; the latter enhanced the expression of the proapoptotic IR-B and MCL-1s rather than the antiapoptotic IR-A and MCL-1L isoforms [35]. Similarly, SRPK1 downregulation also induced hormone-positive breast cancer sensitivity to cisplatin [36]. In contrast, a different SRPK1-driven oncogenic mechanism was noted in BLBC. Van Roosmalen et al. reported that reduced SRPK1 expression suppressed the migration of BLBC cells in vitro and breast cancer metastasis to the lungs in vivo through interacting with the NF-κB (nuclear factor kappa-light-chain-enhancer of activated B cells) pathway [33]. All in all, SRPK1 downregulation enhances apoptosis and sensitivity to chemotherapy in hormone positive, while it suppresses migration and metastasis in BLBC preclinical models.

Likewise, SRPK1-driven alternative splicing is also vital in prostate carcinogenesis and particularly affects VEGF-induced angiogenesis. SRPK1 reduced expression—induced either through gene knockdown or the SRPK1 inhibitor SPHINX—suppressed angiogenesis thus tumor growth in vivo through a switch in VEGF alternative splicing. In particular, expression of the antiangiogenic VEGF165b splicing isoform was favored over the proangiogenic VEGF165. Of interest, SRPK1 downregulation did not alter other key oncogenic processes in vitro such as proliferation, migration, and invasion [26]. To summarize, targeting SRPK1 could treat prostate cancer by suppressing the VEGF-induced angiogenesis.

Several oncogenic pathways and processes were reported to be deregulated after targeting SRPK1 in colorectal cancer. First, SRPK1 downregulation suppressed proliferation, migration, and invasion by interacting with the microRNA miR-216b or the long noncoding RNA MALAT1 (metastasis-associated lung adenocarcinoma transcript 1) [39,63]. Second, like prostate cancer, it also suppressed angiogenesis through a switch in VEGF alternative splicing resulting in higher expression of the VEGF165b isoform [62]. Third, it enhanced apoptosis and resistance to chemotherapy [36,38,61]. Of interest, deregulated alternative splicing was also found to play a role in colorectal carcinogenesis. In the case of the GTPase Rac1 (Ras-related C3 botulinum toxin substrate 1), it resulted in the expression of the isoform Rac1b which sustains tumor survival; SRPK1 downregulation was tumor suppressive through downregulating the expression of Rac1b [60]. Likewise, deregulated alternative splicing of SLC39A14 (zinc transporter ZIP14) resulted in low 4A/4B exon ratio both in precursor lesion (adenoma) and colorectal cancer, when it was high in normal colonic tissue. SRPK1 inhibition increased the 4A/4B exon ratio in colorectal cancer cell lines [31].

Concerning other epithelial cancer types, SRPK1 downregulation suppressed migration and invasion in skin basal cell carcinoma [64]. It also suppressed proliferation, migration, and invasion in stomach cancer preclinical models through various mechanisms, including interactions with the AKT, ERK (extracellular signal regulated kinase) and small nucleolar RNA-mediated pathways, also the microRNA miR-126 [41,42,43,44]. In liver cancer, it suppressed proliferation, migration, and invasion [45,47]. In esophageal cancer, it suppressed proliferation, migration, invasion and enhanced apoptosis [48], while in pancreatic cancer it suppressed proliferation and also enhanced apoptosis and sensitivity to chemotherapy [22]. In kidney cancer, SRPK1 low expression suppressed proliferation, migration, and invasion [50]. Lastly, targeting SRPK1 suppressed proliferation, migration, and invasion whilst it enhanced apoptosis and sensitivity to chemotherapy in ovarian cancer preclinical models [53,54]. Of interest, an older study had suggested that SRPK1 inhibition enhances resistance, rather than sensitivity, to chemotherapy [71].

Similar to prostate and colorectal cancer, the SRPK1/SRSF1-driven VEGF alternative splicing also plays a key role in the regulation of angiogenesis in melanoma. Gammons et al. noted that SRPK1 downregulation (achieved either through gene knockdown or the SRPK1 inhibitor SPRIN340) suppressed angiogenesis by decreasing the expression of proangiogenic VEGF165; of interest, it did not have a significant effect on proliferation [72]. Apart from melanoma tumor cells, VEGF alternative splicing is critical in regulating the oncogenic role of tumor-associated endothelial cells, just like in NSCLC. Low expression of SRPK1 resulted in a shift towards the expression of the antiangiogenic VEGF120, suppressing angiogenesis in vivo [59].

In AML (acute myeloid leukemia), SRPK1 low expression suppressed proliferation while it enhanced cell cycle arrest, apoptosis and survival. SRPK1-mediated aberrant alternative splicing was implicated in the pathogenesis of AML and its inhibition switched BRD4 (bromodomain-containing protein 4) splicing, favoring the production of its long isoform. Of interest, SRPK1 pharmacologic inhibition with SPHINX31 did not impact normal hematopoiesis in the long term [65]. In other studies, SRPK1 downregulation suppressed proliferation and enhanced apoptosis in various leukemia types [66,67,68]. Siqueira et al. also noted that the effect on apoptosis took place in a synergistic fashion with chemotherapy [67].

The most conflicting results from SRPK1 downregulation were derived from glioma studies. Although it suppressed proliferation, migration, invasion, angiogenesis, and cell cycle progression, SRPK1 downregulation also suppressed apoptosis and at the same time induced resistance to chemotherapy in preclinical glioma studies [51,52,69,70]. Therefore, SRPK1 inhibition would require more caution if applied in brain cancers, as it could result in both tumor-suppressive and -promotive effects.

## 4. Discussion and Future Perspectives

SRPK1 is upregulated in the majority of human cancers, where its overexpression was associated with higher TNM staging, grading, and shorter survival in various studies that tested clinical samples (Table 1). Therefore, SRPK1 has the potential to be used as a prognostic biomarker in common malignancies (Table 1) [28], especially the epithelial cancers (e.g., lung, breast, prostate, colorectal, stomach, liver, and esophageal). Apart from cancers themselves, it was also overexpressed in the precursor lesions of prostatic, colorectal, and pancreatic cancers; this suggests that SRPK1 could be involved in early oncogenic steps of these epithelial malignancies [22,26,31]. There were only rare studies that challenged the aforementioned link between SRPK1 expression and cancer promotion; characteristically, these studies investigated non-epithelial cancers. Wu et al. correlated high SRPK1 expression with lower grading in gliomas [51], while Krishnakumar et al. correlated low SRPK1 with higher staging in retinoblastomas, especially the ones that recurred or metastasized [55]. In conclusion, current evidence suggests a prognostic role of SRPK1 in cancer.

Contrary to SRPK1, the other two kinases of the SRPK family, SRPK2 and SRPK3, are much less studied. In normal histology, both SRPK2 and SRPK3 show a tissue-specific expression pattern and, whereas the former is overexpressed in the brain [74], the latter is highly expressed in the skeletal and cardiac muscles [75,76]. Concerning cancer tissues, SRPK2 was upregulated in colon cancer [77], prostate cancer [78], NSCLC [56,79], pancreatic cancer [80], and acute myeloid leukemia [81]. In addition, SRPK2 overexpression was associated with poor survival in NSCLC and pancreatic cancer [56,79,80], also with grading and staging in prostate cancer [78]. In contrast, SRPK3 was downregulated in rhabdomyosarcoma, a mesenchymal pediatric cancer [82].

**Figure 2:**
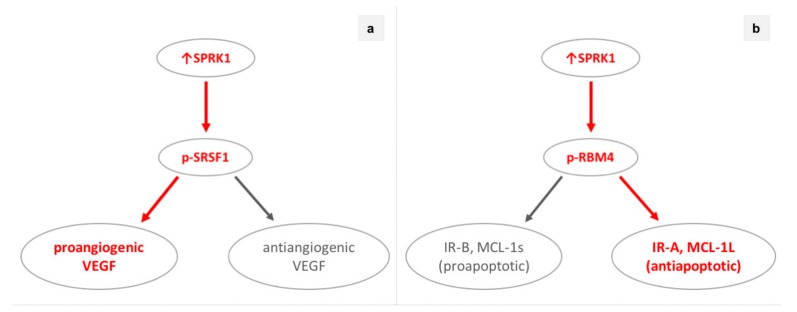
SRPK1 (serine-arginine protein kinase 1) overexpression deregulates alternative splicing and promotes cancer through distinct oncogenic processes such as angiogenesis and apoptosis. (**a**) SRPK1 overexpression switches VEGF (vascular endothelial growth factor) alternative splicing towards the proangiogenic VEGF isoform(s) in prostate cancer, colorectal cancer, melanoma, and tumor endothelium. (**b**) SRPK1 overexpression switches IR (insulin receptor) and MCL-1 (myeloid cell leukemia-1) alternative splicing towards the antiapoptotic isoforms IR-A and MCL-1L, respectively, in hormone-positive breast cancer.

Although not yet tested in human trials, evidence from preclinical studies supports a potential role of SRPK1 in cancer treatment. Indeed, SRPK1 presents as an attractive therapeutic target. First, SRPK1 is implicated in the oncogenesis of multiple cancers and has been targeted with promising results, at the preclinical level, in most of them (Table 2, Figure 1). Second, targeting SRPK1 could simultaneously affect diverse oncogenic processes—proliferation, migration, invasion, metastasis, angiogenesis, apoptosis, acquisition of CTC phenotype, and sensitivity to chemotherapy—through various mechanisms depending on the cancer type (Table 2, Figure 1). For instance, deregulation of alternative splicing was reported to impact angiogenesis in lung [59], prostate [26], colorectal cancer [62] and melanoma [72], also apoptosis in breast [35,36], colorectal cancer [36], and leukemia [65,66] (Figure 2). Of interest, apart from cancer cells, SRPK1 inhibition influences tumor microenvironment and more specifically the tumor-associated endothelial cells, suppressing angiogenesis in vivo [59]. In this context, the SRPK1/SRSF1 axis is regulated by WT-1 (Wilms Tumor-1) [59]. WT1 is upregulated in the endothelium of diverse tumors such as lung, breast, pancreatic, ovarian, bladder cancers, and glioblastoma [59,83], while its endothelial-specific knockout suppresses tumor growth and metastasis [84]. As, in contrast to the endothelium, WT-1 suppresses SRPK1 expression in human podocytes, unidentified co-factors of WT1 might be involved in the regulation of the SRPK1/SRSF1 axis [62].

Targeting SRPK1 effect on cancer appears heterogeneous and depends on the oncogenic context. For instance, SRPK1 inhibition suppresses angiogenesis, metastasis, and the acquisition of a CSC phenotype, and also enhances apoptosis in lung cancer [29,30,58,59]. In contrast, it primarily suppresses angiogenesis albeit causes no significant alteration in proliferation, migration, and invasion in prostate cancer [26] (Figure 1). Molecular subtype also seems to play a critical role; SRPK1 inhibition suppresses metastasis in BLBCs [33], while enhancing apoptosis and sensitivity to platinum-based chemotherapy in hormone-positive breast cancers [36].

Various inhibitors have been used in preclinical studies. SRPIN340, a selective SRPK1 and SRPK2 inhibitor, induced the expression of the antiangiogenic isoform VEGF-165b and suppressed angiogenesis in melanoma and colorectal cancer models [62,72]. SRPIN340 also exhibited a cytotoxic effect, whilst it reduced the expression of the proangiogenic VEGF isoform and enhanced apoptosis in various leukemia cell lines [66]. SPHINX, an inhibitor with preference for SRPK1 [85], suppressed angiogenesis and inhibited tumor growth when it was administered in prostate cancer animal models [26]. SPHINX31, an inhibitor highly selective for SRPK1, enhanced cell cycle arrest and apoptosis in AML [65]. Notably, Hatcher et al. developed the first irreversible SRPK inhibitor; SRPKIN-1, which is selective for both SRPK1 and SRPK2, induced the expression of the antiangiogenic isoform VEGF-165b much more efficiently than SRPIN340 [86].

Resistance to chemotherapy, through diverse mechanisms, is a main cause of treatment failure and death in cancer patients [87,88]. Targeting SRPK1 in preclinical models was linked with sensitivity to platinum-based chemotherapy in breast [36], colorectal [36], pancreatic [22], and ovarian cancer [53]. One study by Schenk et al. noted that SRPK1 inhibition enhanced chemoresistance rather than sensitivity in ovarian cancer [71], contradicting with the previously mentioned ones. Of interest, Schenk et al. used the A2780 ovarian cancer cell line for their experiments [71], which was later found not to represent the most common histology type of ovarian cancer, the high-grade serous carcinoma [89,90]. In contrast, the cell line OVCAR3 used by Odunci et al. [53] has been characterized as a TP53-mutant, a high-grade serous carcinoma cell line [91]. In accordance with their ovarian cancer study, Schenk et al. noted that low SRPK1 expression was associated with resistance to chemotherapy in NSGCTs [23]. SRPK1 inhibition was also reported to promote resistance to chemotherapy in gliomas [51,52]. In summary, current evidence points that SRPK1 inhibition promotes sensitivity to chemotherapy in epithelial cancers such as breast, colorectal, pancreatic, and ovarian, while it promotes resistance in gliomas and NSGCTs.

Even if results from preclinical studies are encouraging, SRPK1 has not yet reached clinical trials. Our opinion is that future research on the role of SRPK1 in cancer should first focus more on personalized medicine. More studies that investigate the distinct molecular subtypes of each cancer type (e.g., breast cancer: hormone positive, HER2-enriched, and BLBCs) rather than regard each cancer as single entity, should be performed. In BLBCs, in contrast to hormone and HER2 positive breast cancers, targeted therapies are still ineffective and toxic chemotherapy is the only viable option [16,92]. Van Roosmalen et al. directed their study to the highly aggressive BLBCs [33], setting an example for future research in this field. In addition, although studies that show that SRPK1 downregulation restores sensitivity to platinum-based chemotherapy have been performed [22,36,53], reports on resistance to targeted therapies (e.g., hormone and anti-HER2 therapies in breast cancer) or immunotherapy are still lacking. Cancer stem cells are associated with cancer recurrence, metastasis, and resistance to chemotherapy [7] yet no relevant study, except the one presented by Gong et al. [29], has been performed.

A deeper understanding of the SRPK1 role in oncogenesis and tumor evolution is still needed. For instance, SRPK1 is overexpressed in colorectal, pancreatic, and prostatic precursor lesions [22,26,31]. However, what happens with the precursor lesions of other cancer types? Likewise, we still lack the knowledge about SRPK1 expression status or its interplay with already established oncogenic pathways in metastatic cancers compared to their primary counterparts. Wagner et al. highlighted the link between SRPK1 and tumor associated endothelial cells in lung cancer and melanoma in vivo models [59]. Nevertheless, how does SRPK1 act upon other critical elements of tumor microenvironment such as CAFs or immune cells? Future preclinical studies could also use better models that recapitulate more accurately specific cancer types or their molecular subtypes. PDX (patient-derived xenograft) models could be a promising approach that we have not yet seen in SRPK1-related cancer research except in the study of Tzelepis et al. [65]. In addition, interacting networks affected by SRPK1 downregulation could be better studied using transcriptomics or proteomics accompanied by big data analysis. Lastly, more evidence concerning the side effects of SRPK1 targeting is needed before its potential implementation into clinical trials. Indeed, studies like the one performed by Tzelepis et al., which showed that SRPK1 inhibition does not significantly affect normal hematopoiesis in the long term [65], offer promise.

In conclusion, the presented evidence shows that SRPK1 has a prognostic role in various cancers, while it could pose as an attractive target for cancer therapy. More effective preclinical models (e.g., PDXs) could give a more personalized direction in SRPK1-related cancer research. The goal would be to assess its use as a potential cancer treatment target in specific scenarios, such as treating specific molecular subtypes of diverse cancers or therapy-resistant malignancies.

## Figures and Tables

**Table 1:** Gene expression studies on human material that highlight the prognostic role of SRPK1 in various cancers. Cancers are listed according to their global estimated number of new cases (highest to lowest; GLOBOCAN 2018 estimates) [28].

Cancer type (Primary Location)	Approach Followed (Testing on Histology Samples/Blood; Data Mining)	Level Tested (mRNA; Protein)	Clinical Significance	Reference
Lung	data mining; histology samples	mRNA; protein	↑SRPK1 was found in NSCLC and was associated with higher tumor stage and shorter survival	[29]
	histology samples	mRNA; protein	↑SRPK1 was found in NSCLC	[30]
	data mining	mRNA	↑SRPK1 was found in lung adenocarcinoma (a NSCLC histologic type)	[31]
Breast	histology samples	mRNA; protein	↑SRPK1 was found in breast cancer and was associated with higher tumor stage and shorter survival	[32]
	histology samples; data mining	mRNA; protein	↑SRPK1 was associated with shorter survival in breast cancer and metastatic disease to the lungs and brain	[33]
	data mining	mRNA	↑SRPK1 was associated with shorter survival in HER2-negative breast cancer patients treated with chemotherapy	[34]
	histology samples	protein	↑SRPK1 was found in breast cancer	[35]
	histology samples	protein	↑SRPK1 was found in breast cancer	[22]
	histology samples	protein	↑SRPK1 was found in breast cancer and was associated with higher tumor grade	[36]
Prostate	histology samples	protein	↑SRPK1 was found in prostate cancer and its precursor (PIN)	[26]
	histology samples	protein	↑SRPK1 was found in prostate cancer and was associated with higher stage	[37]
Colorectal	histology samples	protein	↑SRPK1 was found in colon cancer and its precursor (adenoma)	[22]
	histology samples	protein	↑SRPK1 was found in colon cancer and was associated with higher tumor grade	[36]
	histology samples	mRNA; protein	↑SRPK1 was found in colorectal cancer and was associated with higher tumor stage and shorter survival	[38]
	histology samples	mRNA; protein	↑SRPK1 was found in colorectal cancer and was associated with higher tumor stage and shorter survival	[39]
	histology samples	mRNA	↑SRPK1 was found in colorectal cancer and its precursor (adenoma)	[31]
	histology samples	protein	↑SRPK1 and p-AKT were found in colon cancer	[40]
Stomach	data mining	mRNA	↑SRPK1 was found in gastric cancer	[31]
	histology samples	protein	↑SRPK1 was found in gastric cancer and was associated with higher tumor stage and shorter survival	[41]
	histology samples	protein	↑SRPK1 was found in gastric cancer	[42]
	histology samples	mRNA; protein	↑SRPK1 was found in gastric cancer and was associated with higher tumor stage and shorter survival	[43]
	histology samples	protein	↑SRPK1 was found in gastric cancer and was associated with higher tumor grade, stage, and shorter survival	[44]
Liver	histology samples	mRNA; protein	↑SRPK1 was associated with higher stage and shorter survival; SRPK1 expression was inversely correlated with miR-1296 expression	[45]
	histology samples	mRNA; protein	↑SRPK1 was found in liver cancer and was associated with higher stage and shorter survival	[46]
	histology samples	mRNA; protein	↑SRPK1 was found in liver cancer	[47]
Esophagus	histology samples	mRNA; protein	↑SRPK1 was found in esophageal cancer and was associated with higher grade, stage, and shorter survival	[48]
Pancreas	histology samples	protein	↑SRPK1 was found in pancreatic cancer and its precursor (dysplasia)	[22]
Leukemia	blood	mRNA; protein	↑SRPK1 was found in acute ATL	[49]
Kidney	histology samples	protein	↑SRPK1 was found in renal cell carcinoma	[50]
Glioma	histology samples	protein	↑SRPK1 was found in gliomas and was associated with lower rather than higher tumor grade	[51]
	histology samples	protein	↑SRPK1 was found in glioblastomas (Grade IV gliomas) and was associated with shorter survival	[52]
Ovary	histology samples	protein	↑SRPK1 was found in ovarian cancer	[53]
	histology samples	mRNA	↑SRPK1 was found in ovarian cancer	[54]
Testis	histology samples	protein	↓SRPK1 was associated with resistance to chemotherapy in NSGCTs	[23]
Eye	histology samples	protein	↓SRPK1 was found in advanced retinoblastomas, especially the ones that recurred or metastasized	[55]

SRPK1, serine-arginine protein kinase 1; NSCLC, non-small cell lung carcinoma; HER2, human epidermal growth factor receptor 2; PIN, pancreatic intraepithelial neoplasia; miR-1296, microRNA 1296; ATL, adult T-cell leukemia; p-AKT, phosphorylated-protein kinase B; NSGCTs, non-seminomatous germ cell tumors.

**Table 2:** Published preclinical studies (cell lines/in vitro; animal models/in vivo) that summarize the effect of aberrant SRPK1 expression on distinct oncogenic processes (e.g., proliferation, angiogenesis, apoptosis) via a plethora of mechanisms/pathways, highlighting the potential role of SRPK1 as a therapeutic target in various malignancies. Cancers are listed according to their global estimated number of new cases (highest to lowest; GLOBOCAN 2018 estimates) [28].

Cancer Type (Primary Location)	SRPK1 Upregulation (mRNA and/or Protein Levels)	SRPK1 Downregulation (e.g., Gene Silencing; Pharmacologic Inhibition)	SRPK1-Mediated Mechanism(s)/ Pathway(s) Involved	Reference
Lung	↑SRPK1 promoted cell growth, invasion, CSCs aggregation, and the expression of various stem cell markers in vitro; it also promoted tumor growth and tumorigenicity in vivo	↓SRPK1 suppressed cell growth and CSCs in vitro; it also suppressed tumor growth and tumorigenicity in vivo	SRPK1 interacts with the Wnt/β-catenin pathway	[29]
	↑SRPK1 promoted NSCLC cell growth and migration in vitro	↓SRPK1 suppressed cell growth and migration in vitro, also tumorigenicity in vivo	SRPK1 interacts with the β-catenin/TCF pathway	[30]
	↑SRPK1 was found in NSCLC endothelial cell lines and promoted cell growth, migration, and invasion in vitro	↓SRPK1 suppressed cell growth, migration and invasion and induced apoptosis in vitro; it also suppressed tumor growth and metastasis and prolonged survival in vivo	SRPK1 interacts with the β-catenin/TCF pathway and the GSK3-β	[58]
	↑SRPK1 and proangiogenic VEGF were found in tumor endothelial cells in vivo	↓SRPK1 induced the expression of antiangiogenic VEGF in tumor endothelial cells and suppressed angiogenesis in vivo	The SRPK1/SRSF1 axis interacts with WT-1 and regulates VEGF alternative splicing (proangiogenic vs. antiangiogenic isoforms)	[59]
Breast	↑SRPK1 was found in BLBC cell lines	↓SRPK1 suppressed the migration of BLBC cells in vitro and breast cancer metastasis to the lungs in vivo	SRPK1 interacts with the NF-κB pathway	[33]
	↑SRPK1 was found in hormone-positive and triple-negative breast cancer cell lines and promoted the localization of RBM4 in the cytoplasm; this enhanced the expression of the antiapoptotic IR-A and MCL-1L isoforms in hormone-positive breast cancer cell lines	↓SRPK1 in hormone-positive breast cancer cells promoted apoptosis by reducing the phosphorylation of RBM4 and restoring its localization in the nucleus; this enhanced the expression of the proapoptotic IR-B and MCL-1s isoforms	SRPK1 regulates the alternative splicing of IR and MCL-1 by modulating the localization of RBM4 inside the cell (cytoplasm vs. nucleus)	[35]
	↑SRPK1 was found in hormone-positive and triple-negative breast cancer cell lines	↓SRPK1 induced apoptosis and sensitivity to chemotherapy in hormone-positive breast cancer cells	SRPK1 interacts with the AKT and MAPK pathways and modulates MAP2K2 alternative splicing	[36]
	↑SRPK1 was found in breast cancer cell lines			[32]
Prostate	↑SRPK1 was found in prostate cancer cell lines	↓SRPK1 suppressed angiogenesis (as shown by the reduced MVD) and thus tumor growth in vivo by inducing the expression of the antiangiogenic VEGF-165b	The SRPK1/SRSF1 axis regulates VEGF-A alternative splicing (proangiogenic vs. antiangiogenic VEGF isoforms)	[26]
Colorectal	↑SRPK1 was found in colorectal cancer cell lines	↓SRPK1 induced apoptosis and sensitivity to chemotherapy in vitro	SRPK1 interacts with the AKT and MAPK pathways and modulates MAP2K2 alternative splicing	[36]
	↑SRPK1 was found in colorectal cancer cell lines and induced proliferation in vitro	↓SRPK1 suppressed growth, angiogenesis, migration and induced apoptosis in vitro		[38]
	↑SRPK1 was found in colorectal cancer cell lines	↓SRPK1 suppressed proliferation, migration and invasion in vitro	SRPK1 interacts with the miR-216b	[39]
		↓SRPK1 suppressed the SRSF-1 dependent expression of Rac1b isoform	The SRPK1/SRSF1 axis regulates Rac1 alternative splicing	[60]
	↑SRPK1 was found in colorectal cancer cell lines	↓SRPK1 increased the 4A/4B exon ratio of SLC39A14 in vitro	The SRPK1/SRSF1 axis interacts with the Wnt pathway and regulates SLC39A14 alternative splicing	[31]
	↑SRPK1 was found in the chemoresistant colorectal cancer cell lines	↓SRPK1 was found in the chemosensitive colorectal cancer cell lines	SRPK1 regulates the response to chemotherapy in colorectal cancer cell lines	[61]
		↓SRPK1 induced expression of the antiangiogenic VEGF-165b and suppressed angiogenesis in vitro and in vivo	SRPK1 regulates VEGF alternative splicing	[62]
		↓SRPK1 suppressed proliferation, migration and invasion in vitro	SRPK1 interacts with the long noncoding RNA MALAT1	[63]
Skin (non-melanoma)	↑SRPK1 abolished the suppression of migration, invasion, and EMT caused by SOX2 knockdown in BCC cell lines	↓SRPK1 suppressed migration, invasion, and EMT in vitro	The SRPK1-mediated PI3K/AKT pathway interacts with SOX2	[64]
Stomach	↑SRPK1 was found in gastric cancer cell lines and promoted proliferation and invasion in vitro	↓SRPK1 suppressed proliferation and invasion in vitro	SRPK1 interacts with the AKT and ERK pathways	[41]
	↑SRPK1 promoted proliferation in vitro and in vivo	↓SRPK1 suppressed proliferation both in vitro and in vivo	SRPK1 interacts with the small nucleolar RNA-mediated pathways	[42]
	↑SRPK1 was found in gastric cancer cell lines and promoted proliferation, migration, and invasion in vitro	↓SRPK1 suppressed proliferation, migration, and invasion in vitro	SRPK1 interacts with miR-126	[43]
	↑SRPK1 was found in gastric cancer cell lines	↓SRPK1 abolished the IGF-1-mediated expression of EMT markers, induced cell cycle arrest and suppressed migration and invasion in vitro	The IGF-1/SRPK1 pathway regulates EMT	[44]
Liver	↑SRPK1 promoted migration, invasion, and EMT in vitro; SRPK1 expression was inversely correlated with miR-1296 in vivo and in vitro	↓SRPK1 suppressed migration and invasion in vitro	The SRPK1-induced PI3K/AKT pathway interacts with miR-1296	[45]
	↑SRPK1 was found in hepatocellular cancer cell lines			[46]
	↑SRPK1 was found in hepatocellular cancer cell lines and promoted proliferation	↓SRPK1 suppressed proliferation in vitro and growth in vivo and in vitro	SRPK1 interacts with the PI3K/AKT pathway	[47]
Esophagus	↑SRPK1 was found in esophageal cancer cell lines and promoted proliferation	↓SRPK1 suppressed proliferation, migration, and invasion and enhanced apoptosis in vitro also suppressed tumor growth in vivo	SRPK1 interacts with the TGF-β pathways	[48]
Pancreas	↑SRPK1 was found in pancreatic cancer cell lines	↓SRPK1 suppressed proliferation and enhanced apoptosis and sensitivity to chemotherapy in vitro	SRPK1 interacts with SR proteins and regulates apoptosis	[22]
			SRPK1 interacts with the AKT and MAPK pathways	[36]
Blood (Leukemia)		↓SRPK1 suppressed proliferation in vitro and tumor growth in vivo, while it enhanced cell cycle arrest, apoptosis and prolonged the survival of animal models in MLL-rearranged AML; ↓SRPK1 switched BRD4 splicing, favoring the production of its long isoform	SRPK1 modulates the alternative splicing of BRD4, MYB, and MED24	[65]
	↑SRPK1 was found in various myeloid and lymphoid leukemia cell lines	↓SRPK1 was cytotoxic and enhanced apoptosis in vitro	SRPK1 interacts with the SR proteins and modulates the expression and splicing of MAP2Ks, VEGF, and FAS	[66]
		↓SRPK1 suppressed proliferation while it enhanced autophagy and apoptosis in a synergistic fashion with chemotherapy in vitro	SRPK1 interacts with SR proteins and modulates the expression of MAP2Ks and VEGF and the splicing of RON	[67]
		↓SRPK1 suppressed proliferation and enhanced apoptosis in vitro in CML	SRPK1 interacts with the PARP-caspase-3 pathway	[68]
Kidney	↑SRPK1 was found in renal cancer cell lines	↓SRPK1 suppressed proliferation, migration, and invasion in vitro, also tumor growth in vivo	SRPK1 interacts with PI3K/AKT pathway	[50]
Brain (Glioma)	↑SRPK1 was found in glioma cell lines	↓SRPK1 suppressed proliferation, migration, and invasion of glioma cells in vitro while it induced resistance to chemotherapy		[51]
		↓SRPK1 suppressed tumor growth and apoptosis in vivo, also angiogenesis in vitro and in vivo	The plexin-B1/SRPK1 pathway regulates cell motility, apoptosis, and angiogenesis	[69]
	↑SRPK1 was found in glioma cell lines	↓SRPK1 suppressed cell viability and response to chemotherapy in vitro		[52]
	↑SRPK1 was found in glioma cell lines	↓SRPK1 suppressed apoptosis in vivo and in vitro, also migration and invasion in vivo	SRPK1 interacts with the PI3K/Akt pathway and modulates the expression of MMP-2 and MMP-9	[70]
Ovary	↑SRPK1 was found in ovarian cancer cell lines	↓SRPK1 suppressed proliferation, migration, and cell cycle progression also enhanced sensitivity to chemotherapy in vitro	SRPK1 interacts with the AKT and MAPK pathways	[53]
	↑SRPK1 was found in ovarian cancer cell lines	↓SRPK1 suppressed proliferation, migration, and invasion while it enhanced apoptosis and sensitivity to chemotherapy in vitro	SRPK1 interacts with the long noncoding RNA UCA1	[54]
		↓SRPK1 enhanced resistance to chemotherapy in vitro		[71]
Skin (Melanoma)	↑SRPK1 was found in melanoma cell lines	↓SRPK1 suppressed angiogenesis (as shown by the reduced MVD) and thus tumor growth in vivo by suppressing the expression of proangiogenic VEGF	The SRPK1/SRSF1 axis regulates VEGF alternative splicing	[72]
	↑SRPK1 and proangiogenic VEGF were found in tumor endothelial cells in vivo	↓SRPK1 suppressed angiogenesis in vivo by enhancing antiangiogenic VEGF expression in tumor endothelial cells	The SRPK1/SRSF1 axis interacts with WT-1 and regulates VEGF splicing	[60]
		↓SRPK1 suppressed migration, invasion, adhesion, and colony formation in vitro, also metastasis in vivo		[73]

SRPK1, serine-arginine protein kinase 1; NSCLC, non-small cell lung carcinoma; CSCs, cancer stem cells; TCF, T-cell factor; GSK3-β, glycogen synthase kinase 3-β; VEGF-A, vascular endothelial growth factor-A; BLBC, basal-like breast cancer; SRSF1, Serine/arginine-rich splicing factor 1; WT-1, Wilms tumor-1; NF-κB, nuclear factor kappa-light-chain-enhancer of activated B cells; IR, insulin receptor; MCL-1, myeloid cell leukemia-1; RBM4, RNA-binding motif protein 4; MAPK, mitogen-activated protein kinase; MAP2K2, mitogen-activated protein kinase kinase 2; MVD, microvascular density; miR-216b, microRNA-216b; Rac1, Ras-related C3 botulinum toxin substrate 1; SLC39A14, zinc transporter ZIP14; MALAT1, metastasis-associated lung adenocarcinoma transcript 1; EMT, epithelial mesenchymal transition; BCC, basal cell carcinoma; PI3K, phosphatidylinositol 3-kinase; ERK, extracellular signal regulated kinase; miR-126, microRNA-126, IGF-1, insulin-like growth factor-1; TGF-β, transforming growth factor-β; SR proteins, serine arginine-rich proteins; BRD4, bromodomain-containing protein 4; MED24, mediator complex subunit 24; MLL, mixed-lineage leukemia; AML, acute myeloid leukemia; RON, Recepteur d’Origine Nantais; PARP, poly (ADP-ribose) polymerase; CML, chronic myeloid leukemia; MMP, metalloproteinase; UCA1, urothelial carcinoma-associated 1.

## References

[1] Hanahan D., Weinberg R.A. (2011). Hallmarks of cancer: The next generation. Cell.

[2] Oltean S., Bates D.O. (2014). Hallmarks of alternative splicing in cancer. Oncogene.

[3] Wang M., Zhao J., Zhang L., Wei F., Lian Y., Wu Y., Gong Z., Zhang S., Zhou J., Cao K. (2017). Role of tumor microenvironment in tumorigenesis. J. Cancer.

[4] Sounni N.E., Noel A. (2013). Targeting the tumor microenvironment for cancer therapy. Clin. Chem..

[5] Fisher R., Pusztai L., Swanton C. (2013). Cancer heterogeneity: Implications for targeted therapeutics. Br. J. Cancer.

[6] Nikas I., Ryu H.S., Theocharis S. (2018). Viewing the Eph receptors with a focus on breast cancer heterogeneity. Cancer Lett..

[7] Prasetyanti P.R., Medema J.P. (2017). Intra-tumor heterogeneity from a cancer stem cell perspective. Mol. Cancer.

[8] Brierley J., O’Sullivan B., Asamura H., Byrd D., Huang S.H., Lee A., Piñeros M., Mason M., Moraes F.Y., Rösler W. (2019). Global Consultation on Cancer Staging: Promoting consistent understanding and use. Nat. Rev. Clin. Oncol..

[9] Amin M.B., Edge S.B., Greene F.L., Byrd D.R., Brookland R.K., Washington M.K., Gershenwald J.E., Compton C.C., Hess K.R., Sullivan D.C. (2018). AJCC Cancer Staging Manual.

[10] Sun Z., Aubry M.-C., Deschamps C., Marks R.S., Okuno S.H., Williams B.A., Sugimura H., Pankratz V.S., Yang P. (2006). Histologic grade is an independent prognostic factor for survival in non-small cell lung cancer: An analysis of 5018 hospital- and 712 population-based cases. J. Thorac. Cardiovasc. Surg..

[11] Rakha E.A., Reis-Filho J.S., Baehner F., Dabbs D.J., Decker T., Eusebi V., Fox S.B., Ichihara S., Jacquemier J., Lakhani S.R. (2010). Breast cancer prognostic classification in the molecular era: The role of histological grade. Breast Cancer Res..

[12] Perou C.M., Sørlie T., Eisen M.B., van de Rijn M., Jeffrey S.S., Rees C.A., Pollack J.R., Ross D.T., Johnsen H., Akslen L.A. (2000). Molecular portraits of human breast tumours. Nature.

[13] Sørlie T., Perou C.M., Tibshirani R., Aas T., Geisler S., Johnsen H., Hastie T., Eisen M.B., van de Rijn M., Jeffrey S.S. (2001). Gene expression patterns of breast carcinomas distinguish tumor subclasses with clinical implications. Proc. Natl. Acad. Sci. USA.

[14] (2012). Cancer Genome Atlas Network Comprehensive molecular portraits of human breast tumours. Nature.

[15] Schnitt S.J. (2010). Classification and prognosis of invasive breast cancer: From morphology to molecular taxonomy. Mod. Pathol..

[16] Badve S., Dabbs D.J., Schnitt S.J., Baehner F.L., Decker T., Eusebi V., Fox S.B., Ichihara S., Jacquemier J., Lakhani S.R. (2011). Basal-like and triple-negative breast cancers: A critical review with an emphasis on the implications for pathologists and oncologists. Mod. Pathol..

[17] Bertucci F., Finetti P., Birnbaum D. (2012). Basal breast cancer: A complex and deadly molecular subtype. Curr. Mol. Med..

[18] Corkery D.P., Holly A.C., Lahsaee S., Dellaire G. (2015). Connecting the speckles: Splicing kinases and their role in tumorigenesis and treatment response. Nucleus.

[19] Bowler E., Oltean S. (2019). Alternative Splicing in Angiogenesis. Int. J. Mol. Sci..

[20] Giannakouros T., Nikolakaki E., Mylonis I., Georgatsou E. (2011). Serine-arginine protein kinases: A small protein kinase family with a large cellular presence. FEBS J..

[21] Das S., Krainer A.R. (2014). Emerging functions of SRSF1, splicing factor and oncoprotein, in RNA metabolism and cancer. Mol. Cancer Res..

[22] Hayes G.M., Carrigan P.E., Beck A.M., Miller L.J. (2006). Targeting the RNA splicing machinery as a novel treatment strategy for pancreatic carcinoma. Cancer Res..

[23] Schenk P.W., Stoop H., Bokemeyer C., Mayer F., Stoter G., Oosterhuis J.W., Wiemer E., Looijenga L.H.J., Nooter K. (2004). Resistance to platinum-containing chemotherapy in testicular germ cell tumors is associated with downregulation of the protein kinase SRPK1. Neoplasia.

[24] Mytilinaios D.G., Tsamis K.I., Nikolakaki E., Giannakouros T. (2012). Distribution of SRPK1 in human brain. J. Chem. Neuroanat..

[25] Patel M., Sachidanandan M., Adnan M. (2019). Serine arginine protein kinase 1 (SRPK1): A moonlighting protein with theranostic ability in cancer prevention. Mol. Biol. Rep..

[26] Mavrou A., Brakspear K., Hamdollah-Zadeh M., Damodaran G., Babaei-Jadidi R., Oxley J., Gillatt D.A., Ladomery M.R., Harper S.J., Bates D.O. (2015). Serine-arginine protein kinase 1 (SRPK1) inhibition as a potential novel targeted therapeutic strategy in prostate cancer. Oncogene.

[27] Chandrashekar D.S., Bashel B., Balasubramanya S.A.H., Creighton C.J., Ponce-Rodriguez I., Chakravarthi B.V.S.K., Varambally S. (2017). UALCAN: A Portal for Facilitating Tumor Subgroup Gene Expression and Survival Analyses. Neoplasia.

[28] Bray F., Ferlay J., Soerjomataram I., Siegel R.L., Torre L.A., Jemal A. (2018). Global cancer statistics 2018: GLOBOCAN estimates of incidence and mortality worldwide for 36 cancers in 185 countries. CA Cancer J. Clin..

[29] Gong L., Song J., Lin X., Wei F., Zhang C., Wang Z., Zhu J., Wu S., Chen Y., Liang J. (2016). Serine-arginine protein kinase 1 promotes a cancer stem cell-like phenotype through activation of Wnt/β-catenin signalling in NSCLC. J. Pathol..

[30] Liu H., Hu X., Zhu Y., Jiang G., Chen S. (2016). Up-regulation of SRPK1 in non-small cell lung cancer promotes the growth and migration of cancer cells. Tumour Biol..

[31] Thorsen K., Mansilla F., Schepeler T., Øster B., Rasmussen M.H., Dyrskjøt L., Karni R., Akerman M., Krainer A.R., Laurberg S. (2011). Alternative splicing of SLC39A14 in colorectal cancer is regulated by the Wnt pathway. Mol. Cell. Proteom..

[32] Li X.-H., Song J.-W., Liu J.-L., Wu S., Wang L.-S., Gong L.-Y., Lin X. (2014). Serine-arginine protein kinase 1 is associated with breast cancer progression and poor patient survival. Med. Oncol..

[33] Van Roosmalen W., Le Dévédec S.E., Golani O., Smid M., Pulyakhina I., Timmermans A.M., Look M.P., Zi D., Pont C., de Graauw M. (2015). Tumor cell migration screen identifies SRPK1 as breast cancer metastasis determinant. J. Clin. Invest..

[34] Cheng P., Wang Z., Hu G., Huang Q., Han M., Huang J. (2017). A prognostic 4-gene expression signature for patients with HER2-negative breast cancer receiving taxane and anthracycline-based chemotherapy. Oncotarget.

[35] Lin J.-C., Lin C.-Y., Tarn W.-Y., Li F.-Y. (2014). Elevated SRPK1 lessens apoptosis in breast cancer cells through RBM4-regulated splicing events. RNA.

[36] Hayes G.M., Carrigan P.E., Miller L.J. (2007). Serine-arginine protein kinase 1 overexpression is associated with tumorigenic imbalance in mitogen-activated protein kinase pathways in breast, colonic, and pancreatic carcinomas. Cancer Res..

[37] Bullock N., Potts J., Simpkin A.J., Koupparis A., Harper S.J., Oxley J., Oltean S. (2016). Serine-arginine protein kinase 1 (SRPK1), a determinant of angiogenesis, is upregulated in prostate cancer and correlates with disease stage and invasion. J. Clin. Pathol..

[38] Yi N., Xiao M., Jiang F., Liu Z., Ni W., Lu C., Ni R., Chen W. (2018). SRPK1 is a poor prognostic indicator and a novel potential therapeutic target for human colorectal cancer. Onco. Targets. Ther..

[39] Yao Y., Li Q., Wang H. (2018). MiR-216b suppresses colorectal cancer proliferation, migration, and invasion by targeting SRPK1. Onco. Targets. Ther..

[40] Wang P., Zhou Z., Hu A., Ponte de Albuquerque C., Zhou Y., Hong L., Sierecki E., Ajiro M., Kruhlak M., Harris C. (2014). Both decreased and increased SRPK1 levels promote cancer by interfering with PHLPP-mediated dephosphorylation of Akt. Mol. Cell.

[41] Xu X., Wei Y., Wang S., Luo M., Zeng H. (2017). Serine-arginine protein kinase 1 (SRPK1) is elevated in gastric cancer and plays oncogenic functions. Oncotarget.

[42] Li Y., Yu S., Wang X., Ye X., He B., Quan M., Gao Y. (2019). SRPK1 facilitates tumor cell growth via modulating the small nucleolar RNA expression in gastric cancer. J. Cell. Physiol..

[43] Li Q., Wang G., Wang H. (2018). MiR-126 functions as a tumor suppressor by targeting SRPK1 in human gastric cancer. Oncol. Res..

[44] Wang H., Wang C., Tian W., Yao Y. (2017). The crucial role of SRPK1 in IGF-1-induced EMT of human gastric cancer. Oncotarget.

[45] Xu Q., Liu X., Liu Z., Zhou Z., Wang Y., Tu J., Li L., Bao H., Yang L., Tu K. (2017). MicroRNA-1296 inhibits metastasis and epithelial-mesenchymal transition of hepatocellular carcinoma by targeting SRPK1-mediated PI3K/AKT pathway. Mol. Cancer.

[46] Zhang J., Jiang H., Xia W., Jiang Y., Tan X., Liu P., Jia H., Yang X., Shen G. (2016). Serine-arginine protein kinase 1 is associated with hepatocellular carcinoma progression and poor patient survival. Tumour Biol..

[47] Zhou B., Li Y., Deng Q., Wang H., Wang Y., Cai B., Han Z.-G. (2013). SRPK1 contributes to malignancy of hepatocellular carcinoma through a possible mechanism involving PI3K/Akt. Mol. Cell. Biochem..

[48] Ren G., Sheng L., Liu H., Sun Y., An Y., Li Y. (2015). The crucial role of SRPK1 in TGF-β-induced proliferation and apoptosis in the esophageal squamous cell carcinomas. Med. Oncol..

[49] Hishizawa M., Imada K., Sakai T., Ueda M., Hori T., Uchiyama T. (2005). Serological identification of adult T-cell leukaemia-associated antigens. Br. J. Haematol..

[50] Han X., Yang J., Jia Z., Wei P., Zhang H., Lv W., Sun J., Huo Q. (2017). Knockdown of Serine-Arginine Protein Kinase 1 Inhibits the Growth and Migration in Renal Cell Carcinoma Cells. Oncol. Res. Featur. Preclin. Clin. Cancer Ther..

[51] Wu Q., Chang Y., Zhang L., Zhang Y., Tian T., Feng G., Zhou S., Zheng Q., Han F., Huang F. (2013). SRPK1 Dissimilarly Impacts on the Growth, Metastasis, Chemosensitivity and Angiogenesis of Glioma in Normoxic and Hypoxic Conditions. J. Cancer.

[52] Sigala I., Tsamis K.I., Gousia A., Alexiou G., Voulgaris S., Giannakouros T., Kyritsis A.P., Nikolakaki E. (2016). Expression of SRPK1 in gliomas and its role in glioma cell lines viability. Tumour Biol..

[53] Odunsi K., Mhawech-Fauceglia P., Andrews C., Beck A., Amuwo O., Lele S., Black J.D., Huang R.-Y. (2012). Elevated expression of the serine-arginine protein kinase 1 gene in ovarian cancer and its role in Cisplatin cytotoxicity in vitro. PLoS ONE.

[54] Wang F., Zhou J., Xie X., Hu J., Chen L., Hu Q., Guo H., Yu C. (2015). Involvement of SRPK1 in cisplatin resistance related to long non-coding RNA UCA1 in human ovarian cancer cells. Neoplasma.

[55] Krishnakumar S., Mohan A., Kandalam M., Ramkumar H.L., Venkatesan N., Das R.R. (2008). SRPK1: A cisplatin sensitive protein expressed in retinoblastoma. Pediatr. Blood Cancer.

[56] Gout S., Brambilla E., Boudria A., Drissi R., Lantuejoul S., Gazzeri S., Eymin B. (2012). Abnormal expression of the pre-mRNA splicing regulators SRSF1, SRSF2, SRPK1 and SRPK2 in non small cell lung carcinoma. PLoS ONE.

[57] Bullock N., Oltean S. (2017). The many faces of SRPK1. J. Pathol..

[58] Wu F., Li J., Du X., Zhang W., Lei P., Zhang Q. (2017). Chimeric antibody targeting SRPK-1 in the treatment of non-small cell lung cancer by inhibiting growth, migration and invasion. Mol. Med. Rep..

[59] Wagner K.-D., El Maï M., Ladomery M., Belali T., Leccia N., Michiels J.-F., Wagner N. (2019). Altered VEGF Splicing Isoform Balance in Tumor Endothelium Involves Activation of Splicing Factors Srpk1 and Srsf1 by the Wilms’ Tumor Suppressor Wt1. Cells.

[60] Gonçalves V., Henriques A.F.A., Pereira J.F.S., Neves Costa A., Moyer M.P., Moita L.F., Gama-Carvalho M., Matos P., Jordan P. (2014). Phosphorylation of SRSF1 by SRPK1 regulates alternative splicing of tumor-related Rac1b in colorectal cells. RNA.

[61] Plasencia C., Martínez-Balibrea E., Martinez-Cardús A., Quinn D.I., Abad A., Neamati N. (2006). Expression analysis of genes involved in oxaliplatin response and development of oxaliplatin-resistant HT29 colon cancer cells. Int. J. Oncol..

[62] Amin E.M., Oltean S., Hua J., Gammons M.V.R., Hamdollah-Zadeh M., Welsh G.I., Cheung M.-K., Ni L., Kase S., Rennel E.S. (2011). WT1 mutants reveal SRPK1 to be a downstream angiogenesis target by altering VEGF splicing. Cancer Cell.

[63] Hu Z.-Y., Wang X.-Y., Guo W.-B., Xie L.-Y., Huang Y.-Q., Liu Y.-P., Xiao L.-W., Li S.-N., Zhu H.-F., Li Z.-G. (2016). Long non-coding RNA MALAT1 increases AKAP-9 expression by promoting SRPK1-catalyzed SRSF1 phosphorylation in colorectal cancer cells. Oncotarget.

[64] Li Z.-R., Jiang Y., Hu J.-Z., Chen Y., Liu Q.-Z. (2019). SOX2 knockdown inhibits the migration and invasion of basal cell carcinoma cells by targeting the SRPK1-mediated PI3K/AKT signaling pathway. Oncol. Lett..

[65] Tzelepis K., De Braekeleer E., Aspris D., Barbieri I., Vijayabaskar M.S., Liu W.-H., Gozdecka M., Metzakopian E., Toop H.D., Dudek M. (2018). SRPK1 maintains acute myeloid leukemia through effects on isoform usage of epigenetic regulators including BRD4. Nat. Commun..

[66] Siqueira R.P., Barbosa É.A.A., Polêto M.D., Righetto G.L., Seraphim T.V., Salgado R.L., Ferreira J.G., Barros M.V., de Oliveira L.L., Laranjeira A.B. (2015). Potential Antileukemia Effect and Structural Analyses of SRPK Inhibition by N-(2-(Piperidin-1-yl)-5-(Trifluoromethyl)Phenyl)Isonicotinamide (SRPIN340). PLoS ONE.

[67] Siqueira R.P., de Andrade Barros M.V., Barbosa É.d.A.A., Onofre T.S., Gonçalves V.H.S., Pereira H.S., Silva Júnior A., de Oliveira L.L., Almeida M.R., Fietto J.L.R. (2017). Trifluoromethyl arylamides with antileukemia effect and intracellular inhibitory activity over serine/arginine-rich protein kinases (SRPKs). Eur. J. Med. Chem..

[68] Wang H., Ge W., Jiang W., Li D., Ju X. (2018). SRPK1-siRNA suppresses K562 cell growth and induces apoptosis via the PARP-caspase3 pathway. Mol. Med. Rep..

[69] Chang Y., Li L., Zhang L., Guo X., Feng Z., Zhou J., Zhou S., Feng G., Han F., Huang W. (2016). Plexin-B1 indirectly affects glioma invasiveness and angiogenesis by regulating the RhoA/αvβ3 signaling pathway and SRPK1. Tumour Biol..

[70] Chang Y., Wu Q., Tian T., Li L., Guo X., Feng Z., Zhou J., Zhang L., Zhou S., Feng G. (2015). The influence of SRPK1 on glioma apoptosis, metastasis, and angiogenesis through the PI3K/Akt signaling pathway under normoxia. Tumour Biol..

[71] Schenk P.W., Boersma A.W., Brandsma J.A., den Dulk H., Burger H., Stoter G., Brouwer J., Nooter K. (2001). SKY1 is involved in cisplatin-induced cell kill in Saccharomyces cerevisiae, and inactivation of its human homologue, SRPK1, induces cisplatin resistance in a human ovarian carcinoma cell line. Cancer Res..

[72] Gammons M.V., Lucas R., Dean R., Coupland S.E., Oltean S., Bates D.O. (2014). Targeting SRPK1 to control VEGF-mediated tumour angiogenesis in metastatic melanoma. Br. J. Cancer.

[73] Moreira G.A., Lima G.D.d.A., Siqueira R.P., Barros M.V.d.A., Adjanohoun A.L.M., Santos V.C., Barbosa É.d.A., Loterio R.K., de Paiva J.C., Gonçalves V.H.S. (2018). Antimetastatic effect of the pharmacological inhibition of serine/arginine-rich protein kinases (SRPK) in murine melanoma. Toxicol. Appl. Pharmacol..

[74] Wang H.Y., Lin W., Dyck J.A., Yeakley J.M., Songyang Z., Cantley L.C., Fu X.D. (1998). SRPK2: A differentially expressed SR protein-specific kinase involved in mediating the interaction and localization of pre-mRNA splicing factors in mammalian cells. J. Cell Biol..

[75] Nakagawa O., Arnold M., Nakagawa M., Hamada H., Shelton J.M., Kusano H., Harris T.M., Childs G., Campbell K.P., Richardson J.A. (2005). Centronuclear myopathy in mice lacking a novel muscle-specific protein kinase transcriptionally regulated by MEF2. Genes Dev..

[76] Xu Y., Yu W., Xiong Y., Xie H., Ren Z., Xu D., Lei M., Zuo B., Feng X. (2011). Molecular characterization and expression patterns of serine/arginine-rich specific kinase 3 (SPRK3) in porcine skeletal muscle. Mol. Biol. Rep..

[77] Wang J., Wu H.-F., Shen W., Xu D.-Y., Ruan T.-Y., Tao G.-Q., Lu P.-H. (2016). SRPK2 promotes the growth and migration of the colon cancer cells. Gene.

[78] Zhuo Y.J., Liu Z.Z., Wan S., Cai Z.D., Xie J.J., Cai Z.D., Song S.D., Wan Y.P., Hua W., Zhong W.D. (2018). Enhanced expression of SRPK2 contributes to aggressive progression and metastasis in prostate cancer. Biomed. Pharmacother..

[79] Li X., Yang S., Zhang M., Xie S., Xie Z. (2019). Downregulation of SRPK2 promotes cell cycle arrest though E2F1 in non-small cell lung cancer. Eur. J. Histochem..

[80] Wang G., Sheng W., Shi X., Li X., Zhou J., Dong M. (2019). Serine/arginine protein-specific kinase 2 promotes the development and progression of pancreatic cancer by downregulating Numb and p53. FEBS J..

[81] Jang S.-W., Yang S.-J., Ehlén A., Dong S., Khoury H., Chen J., Persson J.L., Ye K. (2008). Serine/arginine protein-specific kinase 2 promotes leukemia cell proliferation by phosphorylating acinus and regulating cyclin A1. Cancer Res..

[82] Zhang M., Zhu B., Davie J. (2015). Alternative splicing of MEF2C pre-mRNA controls its activity in normal myogenesis and promotes tumorigenicity in rhabdomyosarcoma cells. J. Biol. Chem..

[83] Wagner N., Michiels J.F., Schedl A., Wagner K.-D. (2008). The Wilms’ tumour suppressor WT1 is involved in endothelial cell proliferation and migration: Expression in tumour vessels in vivo. Oncogene.

[84] Wagner K.-D., Cherfils-Vicini J., Hosen N., Hohenstein P., Gilson E., Hastie N.D., Michiels J.-F., Wagner N. (2014). The Wilms’ tumour suppressor Wt1 is a major regulator of tumour angiogenesis and progression. Nat. Commun..

[85] Gammons M.V., Fedorov O., Ivison D., Du C., Clark T., Hopkins C., Hagiwara M., Dick A.D., Cox R., Harper S.J. (2013). Topical antiangiogenic SRPK1 inhibitors reduce choroidal neovascularization in rodent models of exudative AMD. Invest. Ophthalmol. Vis. Sci..

[86] Hatcher J.M., Wu G., Zeng C., Zhu J., Meng F., Patel S., Wang W., Ficarro S.B., Leggett A.L., Powell C.E. (2018). SRPKIN-1: A Covalent SRPK1/2 Inhibitor that Potently Converts VEGF from Pro-angiogenic to Anti-angiogenic Isoform. Cell Chem Biol.

[87] Luqmani Y.A. (2005). Mechanisms of drug resistance in cancer chemotherapy. Med. Princ. Pract..

[88] Alfarouk K.O., Stock C.-M., Taylor S., Walsh M., Muddathir A.K., Verduzco D., Bashir A.H.H., Mohammed O.Y., Elhassan G.O., Harguindey S. (2015). Resistance to cancer chemotherapy: Failure in drug response from ADME to P-gp. Cancer Cell Int..

[89] Beaufort C.M., Helmijr J.C.A., Piskorz A.M., Hoogstraat M., Ruigrok-Ritstier K., Besselink N., Murtaza M., van Ĳcken W.F.J., Heine A.A.J., Smid M. (2014). Ovarian cancer cell line panel (OCCP): Clinical importance of in vitro morphological subtypes. PLoS ONE.

[90] Ince T.A., Sousa A.D., Jones M.A., Harrell J.C., Agoston E.S., Krohn M., Selfors L.M., Liu W., Chen K., Yong M. (2015). Characterization of twenty-five ovarian tumour cell lines that phenocopy primary tumours. Nat. Commun..

[91] Bourgeois D.L., Kabarowski K.A., Porubsky V.L., Kreeger P.K. (2015). High-grade serous ovarian cancer cell lines exhibit heterogeneous responses to growth factor stimulation. Cancer Cell Int..

[92] Garrido-Castro A.C., Lin N.U., Polyak K. (2019). Insights into Molecular Classifications of Triple-Negative Breast Cancer: Improving Patient Selection for Treatment. Cancer Discov..

